# Blood pressure and the hypertension care cascade in The Gambia: Findings from a nationwide survey

**DOI:** 10.1111/jch.14806

**Published:** 2024-04-02

**Authors:** Modou Jobe, Islay Mactaggart, Abba Hydara, Min J. Kim, Suzannah Bell, Gaetan Brezesky Kotanmi, Omar Badjie, Andrew M. Prentice, Matthew J. Burton

**Affiliations:** ^1^ Medical Research Council Unit The Gambia London School of Hygiene and Tropical Medicine Fajara The Gambia; ^2^ International Centre for Eye Health London School of Hygiene & Tropical Medicine London UK; ^3^ Sheikh Zayed Regional Eye Care Centre Banjul The Gambia; ^4^ International Statistics and Epidemiology Group Department of Infectious Disease Epidemiology London School of Hygiene and Tropical Medicine London UK; ^5^ Moorfields Eye Hospital NHS Foundation Trust London UK; ^6^ Health Promotion & Education Ministry of Health Banjul The Gambia; ^7^ National Institute for Health Research Biomedical Research Centre for Ophthalmology Moorfields Eye Hospital NHS Foundation Trust London UK

**Keywords:** blood pressure, hypertension, hypertension care cascade, pulse pressure, sub‐Saharan Africa

## Abstract

Community treatment of hypertension in sub‐Saharan Africa is hampered by gaps at several stages of the care cascade. We compared blood pressure (BP) levels (systolic, diastolic and pulse pressures) in four groups of participants by hypertension and treatment status. We conducted a nationally representative survey of adults 35 years and older using a multistage sampling strategy based on the 2013 Gambia Population and Housing Census. The BP measurements were taken in triplicate 5 min apart, and the average of the last two measurements was used for analysis. Systolic and diastolic BP levels and pulse pressure were compared by hypertension status using mean and 95% confidence intervals (CI). 53.1% of the sample were normotensive with mean systolic BP (SBP) of 119.2 mmHg (95% CI, 118.7–119.6) and diastolic BP (DBP) of 78.1 mmHg (77.8–78.3). Among individuals with hypertension, mean SBP was 148.7 mmHg (147.7–149.7) among those unaware of their hypertension, 152.2 mmHg (151.0–153.5) among treated individuals and was highest in untreated individuals at 159.3 mmHg (157.3–161.2). The findings were similar for DBP levels, being 93.9 mmHg (93.4–94.4) among the unaware, 95.1 mmHg (94.4–95.8) among the treated and highest at 99.1 mmHg (98.1–100.2) in untreated participants. SBP and DBP were higher in men, and SBP was as expected higher in those aged ≥55 years. BP level was similar in urban and rural areas. Our data shows high BP levels among participants with hypertension including those receiving treatment. Efforts to reduce the health burden of hypertension will require inputs at all levels of the care cascade.

## 1 INTRODUCTION

Hypertension continues to be a major risk factor for cardiovascular disease and mortality globally.^1^, ^2^, ^3^ It is highly prevalent in low‐ and middle‐income countries especially in sub‐Saharan Africa^4^ and as such, poses significant direct impact on patients, their families and to national health services. The complications of hypertension in these countries are increasingly experienced by younger people who form the productive base of the economy. This can impede economic development and exacerbates poverty by diverting vital economic resources from other areas of national development.^5^, ^6^, ^7^

Hypertension is frequently undetected, and untreated or sub‐optimally treated.^8^ Each of these scenarios is associated with complications. The access and quality of care for hypertension has been widely assessed with the cascade of care framework.^9^, ^10^ This framework provides important information on the prevalence of hypertension, the proportion of hypertensive patients who are aware of their condition, the proportion receiving treatment and those achieving the desired level of blood pressure control when treated.^11^ This framework however does not give insights into blood pressure levels of individuals at different stages of the care cascade.

Elevated blood pressure (BP) levels, especially systolic, are a well‐established factor for predicting cardiovascular risk.^12^, ^13^, ^14^ A recent analysis of global data estimated that at age 50 years, a 20 mmHg increase in systolic blood pressure (SBP) increases the risk of cardiovascular disease by approximately 60% and the risk of dying from all causes by 35%−45%.^3^ Previous studies have found a high prevalence of cardiovascular complications in individuals with hypertension regardless of awareness or treatment status.^10^ However, the extent to which this occurs in sub‐Saharan Africa where hypertension levels are often extremely high is unknown.

In the present study, we assessed and compared BP and pulse pressure levels in normotensive individuals (those with a BP <140/90 mmHg and with no self‐reported history or treatment of hypertension), individuals found with an elevated BP level (≥140/90 mmHg) but no previous diagnosis of hypertension or history of treatment (“unaware”), individuals with self‐reported hypertension who were not receiving treatment (“untreated”), and individuals with self‐reported hypertension who were receiving treatment (“treated”). We further examined this by age group, sex and urban/rural residence among adults aged ≥35 years in The Gambia.

## 2 METHODS

We analyzed data from a non‐communicable disease (NCD) survey embedded in the 2019 Gambia National Eye Health Survey. The detailed study protocol is published elsewhere.^15^ The survey identified a nationally representative sample of adults aged ≥35 years using a multistage sampling strategy based on the 2013 Gambia Population and Housing Census. The census enumeration areas were used as clusters, stratified into urban and rural. The clusters were selected to reflect the regional population using probability proportionate to size sampling methods. In the selected cluster, enumerators listed all eligible participants and then grouped them into segments of 30 participants. A segment was then selected at random by drawing a number out of a hat. Detailed study information was provided to selected participants prior to obtaining a signed or thumb printed informed consent. They were subsequently invited to a central location on a given day for data collection.

### 2.1 Data collection procedures

We collected data electronically using the Open Data Kit (ODK) platform installed on Android tablets. Prior to data collection, staff were trained on study procedures and the questionnaire was pre‐tested in a sample of the general population. Consenting participants were interviewed to collect their socio‐demographic and economic information. Men and women were categorized into five age bands. Level of education was defined according to the highest level attained in either conventional school or the madrassa system, pre‐coded as: pre‐school, madrassa (pre‐school), primary (lower basic), madrassa (lower basic), secondary (upper basic, senior), secondary (madrassa), higher (tertiary, university, college), vocational, non‐standard curriculum. These were further categorized into pre‐school/no school, primary, secondary/vocational, higher, don't know/other, and non‐formal/Quranic. Ethnicity was categorized based on self‐attribution. We recorded marital status as never married, currently married, widowed, or divorced. Data on occupation was obtained in pre‐coded categories as: professional/technical/managerial, clerical, sales and services, skilled manual, unskilled manual, domestic service, agriculture, and other. We further categorized this as unemployed, manual, trades, professional, other and retired/old age. We used socio‐economic data to calculate wealth quintiles using the EquityTool as previously described.^15^, ^16^ We also collected information on smoking and alcohol consumption. We measured height to the nearest 0.1 cm with the participant standing fully erect against a portable stadiometer (Leicester Height Measure, Seca, Hamburg, Germany) and without footwear or headwear. Weight was measured to the nearest 0.01 kg using portable scales (Seca, Hamburg, Germany). Body mass index (BMI) was calculated as weight in kilograms divided by height in metres squared. Based on BMI, participants were categorized as underweight (<18.0  kg/m^2^), normal weight (18.0−24.9 kg/m^2^), overweight (25.0−29.9 kg/m^2^), and obese (≥30.0 kg/m^2^).

BP was measured with the participant seated after resting for at least 10 min, with their arm supported at the level of the heart and resting on a surface. The measurements were conducted in the presence of a study nurse and took place in a quiet area within the central location in the community where data collection took place. Measurement was taken in triplicate using automated OMRON‐Healthcare 10 Series blood pressure monitors (Omron, Kyoto, Japan). The BP measurements were taken five minutes apart, and the average of the last two measurements was used for analysis.

### 2.2 Outcome variables

We classified individuals into four categories as follows: (i) individuals with a normal BP and no self‐reported history or treatment of hypertension (“normotensive,” see definition above); (ii) individuals with an elevated BP but without a self‐reported history of hypertension (“unaware,” see hypertension definition above); (iii) individuals with a self‐reported history of hypertension but not receiving treatment (“untreated”); and (iv) individuals with a self‐reported history of hypertension and receiving treatment (“treated”).

### 2.3 Statistical analysis

This was a nationwide eye health and comorbidities survey where the sample size was calculated to detect disease prevalence as low as 0.5% (blindness) with a 95% confidence level and a margin of error of 0.25%. Given that samples will be selected from clusters of 40 persons each, a design effect of 2.5 was applied, assuming that samples will be moderately clustered with an intraclass correlation coefficient of 0.038. A 20% non‐response/dropout rate was also applied, resulting in the final sample size of 10 800. Further information on sample size calculation is detailed elsewhere.^15^

We compared the sample population with the target population on demographic indicators including sex, age, and cluster. The survey oversampled women compared to men by more than twofold (70.3% women vs. 29.7% men). It also showed that selection probabilities were lower than expected in several age groups (5‐year age band) and in clusters. Poststratification sample weights were calculated to account for the disproportionate sampling of one group over another. The dataset was weighted to generalize the findings to the population of The Gambia according to the 2013 Population and Housing Census.^17^ These weights were adjusted to ensure that sex and age (5‐year age band) match those of the standard population. These weights were then multiplied with the cluster selection probabilities, provided that each cluster represents about 30 individuals.

We summarized the demographic characteristics of participants overall and by sex and location. We considered age of participants first as a continuous variable which was summarized using mean (standard deviation, SD). We then categorized age into 5 deciles and summarized it, as well as other categorical variables using count and proportion (column percentage). After weighting, we rounded up to the nearest integer for absolute frequencies. Percentages were calculated to one decimal place. Variables were then summarized by participant hypertension status where we used row percentages. Finally, we compared BP levels by hypertension status group using mean and 95% confidence interval (CI). BP levels of groups whose 95% CIs did not overlap were regarded to be significantly different. Data analyses were conducted using R (version 4.1.1).

## 3 RESULTS

We enumerated a total of 11 127 in this nationwide survey of whom 9788 (88.0%) took part. In the present analysis, we excluded 600 (6.1%) participants with either missing household data or incomplete data and a further 17 (0.2%) participants with missing hypertension data. A total of 9171 participants were therefore included in the present analysis. Table 1 shows the socio‐demographic characteristics of the participants. Overall, after post‐stratification weighting, we achieved approximately equal proportions of men (4589, 50.0%) and women (4582, 50.0%). More than half (54.1%) of the participants were urban residents. The mean age was 49.5 years (SD, 13) and was similar between men and women. The dominant age group was 35–44 years accounting for 42.5% of the men and 44.5% of the women; a similar proportion in urban and rural areas. Overall, about half (48.9%) of the participants had non‐formal education; more than half (54.6%) of women compared to men (43.3%). Participants with at least primary education accounted for 31.9% of the participants overall; 41.1% of the men and 22.4% of the women. The Mandinka ethnic group was the most common representing 37.2% of the sample whilst 22.0% belonged to the Fula ethnic group. Eighty‐five percent of the participants were currently married and only 2.3% were never married. Manual occupation represented about half (49.3%) of the participants. 9.4% of the participants were classified into poorest quintile compared to 27.4% into the richest quintile.

**TABLE 1:** Socio‐demographic characteristics of participants.

	Total			Urban (*N* = 4966)		Rural (*N* = 4205)	
Characteristics	All (9171)	M (*N* = 4589)	W (*N* = 4582)	M (*N* = 2302)	W (*N* = 2664)	M (*N* = 2286)	W (*N* = 1919)
Mean age (SD)	49.5 (13.0)	49.7 (12.7)	49.3 (13.3)	50 (12.8)	49.3 (13.3)	49.3 (12.6)	49.3 (13.3)
Age group							
35–44	3992 (43.5)	1951 (42.5)	2041 (44.5)	974 (42.3)	1174 (44.1)	977 (42.7)	867 (45.2)
45–54	2454 (26.8)	1252 (27.3)	1202 (26.2)	593 (25.8)	718 (27.0)	659 (28.8)	484 (25.2)
55–64	1349 (14.7)	714 (15.6)	635 (13.9)	386 (16.8)	383 (14.4)	328 (14.3)	252 (13.1)
65–74	808 (8.8)	414 (9.0)	393 (8.6)	218 (9.5)	208 (7.8)	196 (8.6)	185 (9.6)
75+	568 (6.2)	257 (5.6)	311 (6.8)	131 (5.7)	181 (6.8)	126 (5.5)	131 (6.8)
Level of education							
Pre‐school/no school	1612 (17.6)	669 (14.6)	942 (20.6)	310 (13.5)	558 (20.9)	359 (15.7)	385 (20.1)
Primary	979 (10.7)	511 (11.1)	468 (10.2)	276 (12.0)	351 (13.2)	235 (10.3)	117 (6.1)
Secondary/vocational)	1527 (16.7)	1043 (22.7)	484 (10.6)	706 (30.7)	396 (14.9)	337 (14.7)	88 (4.6)
Higher	410 (4.5)	336 (7.3)	74 (1.6)	278 (12.1)	67 (2.5)	59 (2.6)	7 (0.4)
Don't know/other	155 (1.7)	43 (0.9)	111 (2.4)	6 (0.3)	47 (1.8)	38 (1.7)	65 (3.4)
Non‐formal/Quranic	4488 (48.9)	1986 (43.3)	2502 (54.6)	727 (31.6)	1246 (46.8)	1259 (55.1)	1257 (65.5)
Ethnicity							
Mandinka/Jahanka	3413 (37.2)	1568 (34.2)	1845 (40.3)	949 (41.2)	1210 (45.4)	619 (27.1)	635 (33.1)
Wollof	1358 (14.8)	723 (15.8)	635 (13.9)	244 (10.6)	281 (10.6)	479 (21.0)	354 (18.4)
Jola/Karoninka	1026 (11.2)	496 (10.8)	530 (11.6)	284 (12.3)	368 (13.8)	211 (9.2)	162 (8.4)
Fula/Tukulor/Lorobo	2019 (22.0)	1157 (25.2)	862 (18.8)	493 (21.4)	411 (15.4)	664 (29.0)	451 (23.5)
Sarahuleh	692 (7.5)	311 (6.8)	380 (8.3)	120 (5.2)	156 (5.9)	191 (8.4)	225 (11.7)
Others	664 (7.2)	334 (7.3)	330 (7.2)	211 (9.2)	237 (8.9)	122 (5.3)	92 (4.8)
Marital status							
Never married	208 (2.3)	177 (3.9)	31 (0.7)	122 (5.3)	26 (1.0)	56 (2.4)	5 (0.3)
Married/living together	7804 (85.1)	4314 (94)	3490 (76.2)	2113 (91.8)	1981 (74.4)	2201 (96.2)	1510 (78.7)
Widowed	988 (10.8)	29 (0.6)	959 (20.9)	17 (0.7)	569 (21.4)	12 (0.5)	390 (20.3)
Divorced/separated	171 (1.9)	69 (1.5)	102 (2.2)	50 (2.2)	88 (3.3)	18 (0.8)	14 (0.7)
Occupation							
Unemployed	1049 (11.4)	364 (7.9)	685 (14.9)	264 (11.5)	477 (17.9)	100 (4.4)	208 (10.8)
Manual	4518 (49.3)	1953 (42.6)	2565 (56.0)	436 (18.9)	1088 (40.9)	1518 (66.4)	1476 (77.0)
Trade	2565 (28.0)	1489 (32.5)	1076 (23.5)	1107 (48.1)	928 (34.8)	382 (16.7)	148 (7.7)
Professional	646 (7.0)	559 (12.2)	87 (1.9)	371 (16.1)	75 (2.8)	188 (8.2)	12 (0.6)
Other	163 (1.8)	146 (3.2)	17 (0.4)	71 (3.1)	12 (0.5)	75 (3.3)	5 (0.3)
Retired	229 (2.5)	77 (1.7)	152 (3.3)	53 (2.3)	83 (3.1)	24 (1.0)	69 (3.6)
Wealth quintile							
1 (Poorest)	862 (9.4)	478 (10.4)	385 (8.4)	43 (1.9)	28 (1.1)	434 (19.0)	357 (18.6)
2	1419 (15.5)	795 (17.3)	624 (13.6)	151 (6.6)	125 (4.7)	644 (28.2)	500 (26.1)
3	2238 (24.4)	1176 (25.6)	1062 (23.2)	211 (9.2)	201 (7.5)	966 (42.3)	861 (44.9)
4	2140 (23.3)	1039 (22.6)	1101 (24.0)	797 (34.6)	899 (33.7)	242 (10.6)	201 (10.5)
5 (Richest)	2511 (27.4)	1100 (24.0)	1411 (30.8)	1100 (47.8)	1411 (53.0)	0 (0)	0 (0)

*Note*: Data are in *n* (%).

Abbreviations: M, men; SD, standard deviation; W, women.

Table 2 shows the characteristics of the population by hypertension status. The majority (53.1%) of the participants were normotensive whilst 21.5% were unaware of their hypertension, 19.6% were receiving treatment and 5.8% were untreated. Whilst the proportion of men was higher in the normotensive and unaware categories, 64.2% of treated and 56.3% of untreated individuals were women.

**TABLE 2:** Socio‐demographic characteristic of participants by hypertension status.

		Hypertension status				
Characteristics	All	Normotensive (*N* = 4869)	Untreated (*N* = 535)	Treated (*N* = 1794)	Unaware (*N* = 1973)	Missing
Sex						
Men	4589 (50)	2542 (52.2)	234 (43.7)	643 (35.8)	1168 (59.2)	0
Women	4582 (50)	2327 (47.8)	301 (56.3)	1151 (64.2)	805 (40.8)	
Location						
Urban	4966 (54.1)	2664 (54.7)	313 (58.5)	949 (52.9)	1047 (53.1)	0
Rural	4205 (45.9)	2205 (45.3)	222 (41.5)	844 (47.1)	926 (46.9)	
Age (years)						
Mean (SD)	49.5 (13)	45.2 (10.6)	54.5 (13)	56.6 (13.7)	52.1 (13.6)	0
Age group						
35–44	3992 (43.5)	2780 (57.1)	138 (25.8)	371 (20.7)	710 (36)	0
45–54	2454 (26.8)	1281 (26.3)	155 (29)	496 (27.6)	519 (26.3)	
55–64	1349 (14.7)	476 (9.8)	114 (21.3)	405 (22.6)	357 (18.1)	
65–74	808 (8.8)	204 (4.2)	83 (15.5)	298 (16.6)	222 (11.3)	
75+	568 (6.2)	127 (2.6)	45 (8.4)	224 (12.5)	165 (8.4)	
Level of education						
Pre‐school/no school	1612 (17.6)	791 (16.2)	49 (9.2)	437 (24.4)	336 (17)	0
Primary	979 (10.7)	603 (12.4)	43 (8)	146 (8.1)	189 (9.6)	
Secondary/vocational)	1527 (16.7)	951 (19.5)	78 (14.6)	189 (10.5)	309 (15.7)	
Higher	410 (4.5)	258 (5.3)	17 (3.2)	46 (2.6)	90 (4.6)	
Don't know/other	155 (1.7)	65 (1.3)	9 (1.7)	37 (2.1)	40 (2)	
Non‐formal/Quranic	4488 (48.9)	2200 (45.2)	339 (63.4)	939 (52.3)	1008 (51.1)	
Ethnicity						
Mandinka/Jahanka	3413 (37.2)	1814 (37.3)	203 (38)	696 (38.8)	703 (35.6)	0
Wollof	1358 (14.8)	750 (15.4)	78 (14.6)	257 (14.3)	272 (13.8)	
Jola/Karoninka	1026 (11.2)	569 (11.7)	61 (11.4)	160 (8.9)	236 (12)	
Fula/Tukulor/Lorobo	2019 (22)	1098 (22.6)	118 (22.1)	349 (19.5)	455 (23)	
Sarahuleh	692 (7.5)	297 (6.1)	36 (6.7)	189 (10.5)	171 (8.7)	
Others	664 (7.2)	341 (7)	38 (7.1)	142 (7.9)	137 (6.9)	
Marital status						
Never married	208 (2.3)	148 (3)	3 (0.6)	11 (0.6)	47 (2.4)	0
Married/living together	7804 (85.1)	4355 (89.4)	434 (81.1)	1363 (76)	1657 (84)	
Widowed	988 (10.8)	272 (5.6)	90 (16.8)	394 (22)	226 (11.5)	
Divorced/separated	171 (1.9)	94 (1.9)	8 (1.5)	26 (1.4)	42 (2.1)	
Occupation						
Unemployed	1049 (11.4)	340 (7)	126 (23.6)	324 (18.1)	256 (13)	0
Manual	4518 (49.3)	2436 (50)	225 (42.1)	894 (49.8)	960 (48.7)	
Trade	2565 (28)	1547 (31.8)	151 (28.2)	357 (19.9)	517 (26.2)	
Professional	646 (7)	404 (8.3)	22 (4.1)	78 (4.3)	144 (7.3)	
Other	163 (1.8)	99 (2)	2 (0.4)	29 (1.6)	34 (1.7)	
Retired	229 (2.5)	43 (0.9)	9 (1.7)	112 (6.2)	62 (3.1)	
Wealth quintile						
1 (Poorest)	862 (9.4)	450 (9.2)	53 (9.9)	160 (8.9)	198 (10)	0
2	1419 (15.5)	787 (16.2)	90 (16.9)	246 (13.7)	296 (15)	
3	2238 (24.4)	1179 (24.2)	95 (17.8)	475 (26.5)	484 (24.5)	
4	2140 (23.3)	1131 (23.2)	141 (26.4)	426 (23.7)	443 (22.4)	
5 (Richest)	2511 (27.4)	1322 (27.2)	155 (29)	487 (27.1)	553 (28)	
BMI (kg/m^2^)						
Mean (SD)	24.2 (5.1)	23.5 (4.7)	25.9 (5.4)	25.8 (5.7)	24.0 (4.9)	465
BMI categories						
Underweight	623 (7.2)	394 (8.4)	24 (4.9)	91 (5.5)	112 (6.0)	465
Normal	4893 (56.2)	2820 (60)	225 (45.6)	726 (44.0)	1119 (60.1)	
Overweight	2143 (24.6)	1067 (22.7)	141 (28.6)	487 (29.5)	451 (24.2)	
Obese	1047 (12.0)	419 (8.9)	103 (20.9)	346 (21.0)	181 (9.7)	

*Note*: Data are in *n* (%).

Participants with normotension were younger with a mean age of 45.2 years (SD, 10.6) compared to the other groups. While most participants aged between 35 and 54 years did not have hypertension, only the minority of those aged 55 years and above were normotensive. For all categories of hypertension, the majority (61.4%) did not attain primary level of education or had non‐formal education. Similarly, those in active occupation (manual occupation, trader, professional) formed the majority in all categories.

The mean SBP and diastolic BP (DBP) respectively in the overall population was 134.4 mmHg (95% CI, 133.7–135.1) and 86.1 mmHg (95% CI, 85.7–86.4). However, those without hypertension had a mean SBP of 119.2 mmHg (95% CI, 118.7–119.6) and a mean DBP of 78.1 mmHg (95% CI, 77.8–78.3) (Table 3). The mean SBP was unsurprisingly highest among the untreated at 159.3 mmHg (95% CI, 157.3–161.2) followed by those receiving treatment at 152.2 mmHg (95% CI, 151.0–153.5) and was 148.7 mmHg (95% CI, 147.7–149.7) among those unaware of their hypertension. A similar pattern was observed for DBP being 99.1 mmHg (95% CI, 98.1–100.2) in the untreated, 95.1 mmHg (95% CI, 94.4–95.8) in the treated and lowest at 93.9 mmHg (95% CI, 93.4–94.4) among those unaware of their hypertension. Generally, and irrespective of hypertension status, men had higher SBP but not DBP. As for location, except for those unaware of their hypertension, the average SBP was similar across urban and rural areas. For those who were unaware of their hypertension, SBP was higher in rural versus urban areas. Irrespective of hypertension status, average DBP was similar between rural and urban areas (Table 3).

**TABLE 3:** Mean blood pressure (mmHg) and 95% confidence interval of the mean according to hypertension status by sex and location.

	All	Normal^a^	Untreated	Treated	Unaware
	Systolic blood pressure, mmHg (95% confidence interval)				
Overall	134.4 (133.7–135.1)	119.2 (118.7–119.6)	159.3 (157.3–161.2)^b^	152.2 (151.0–153.5)^b^	148.7 (147.7–149.7)^b^
Sex					
Men	135.5 (134.4–136.5)	121.4 (120.7–122.0)	162.4 (158.7–166.2)^b^	155.9 (153.3–158.4)^b^	149.5 (148.2–150.8)^b^
Women	133.2 (132.5–134.0)	116.7 (116.3–117.2)	156.8 (154.7–159.0)^b^	150.2 (148.9–151.6)^b^	147.5 (146.2–148.8)^b^
Location					
Urban	133.8 (133.0–134.7)	119.1 (118.5–119.6)	158.7 (156.3–161.0)^b^	152.4 (150.6–154.1)^b^	147.2 (146.0–148.4)^b^
Rural	135 (133.8–136.1)	119.2 (118.6–119.9)	160.1 (156.7–163.5)^b^	152.1 (150.2–154.0)^b^	150.4 (148.9–151.9)^b^
	Diastolic blood pressure, mmHg (95% confidence interval)				
Overall	86.1 (85.7–86.4)	78.1 (77.8–78.3)	99.1 (98.1–100.2)^b^	95.1 (94.4–95.8)^b^	93.9 (93.4–94.4)^b^
Sex					
Men	85.7 (85.1–86.3)	78 (77.6–78.4)	100.1 (98.1–102.1)^b^	96.5 (95.0–97.9)^b^	93.7 (93.0–94.5)^b^
Women	86.4 (86.0–86.8)	78.2 (77.9–78.4)	98.4 (97.4–99.4)^b^	94.4 (93.7–95.1)^b^	94.2 (93.5–94.8)^b^
Location					
Urban	86.0 (85.5–86.4)	78.2 (77.9–78.6)	99.4 (98.0–100.7)^b^	94.9 (94.0–95.9)^b^	93.6 (92.9–94.3)^b^
Rural	86.2 (85.5–86.8)	77.9 (77.5–78.3)	98.8 (97.2–100.4)^b^	95.3 (94.3–96.4)^b^	94.3 (93.5–95.1)^b^
	Pulse pressure, mmHg (95% confidence interval)				
Overall	48.3 (47.8–48.8)	41.1 (40.7–41.5)	60.1 (58.3–62.0)^b^	57.1 (56.1–58.1)^b^	54.8 (53.9–55.7)^b^
Sex					
Men	49.8 (49.1–50.4)	43.4 (42.8–43.9)	62.3 (59.0–65.7)^b^	59.4 (57.7–61.1)^b^	55.8 (54.6–56.9)^b^
Women	46.8 (46.3–47.4)	38.6 (38.2–38.9)	58.5 (56.4–60.5)^b^	55.9 (54.7–57.0)^b^	53.3 (52.1–54.6)^b^
Location					
Urban	47.9 (47.2–48.5)	40.9 (40.3–41.4)	59.3 (57.2–61.5)^b^	57.4 (56.0–58.8)^b^	53.6 (52.4–54.8)^b^
Rural	48.8 (48.1–49.5)	41.3 (40.8–41.9)	61.3 (58.1–64.5)^b^	56.8 (55.4–58.2)^b^	56.1 (54.8–57.4)^b^

^a^ Normotensive individuals have significantly lower blood pressure compared to the groups with hypertension (untreated, treated an unaware).

^b^ Denotes significant difference in blood pressure among individuals with hypertension (untreated, treated an unaware).

We also compared pulse pressure between groups and found that this was significantly lower in those without hypertension at 41.1 mmHg (95% CI, 40.7–41.5). Hypertensive individuals not receiving treatment had significantly wider pulse pressure at 60.1 mmHg (95% CI, 58.3–62.0) compared to the other groups with hypertension. This was followed by the treated group and was lowest in those unaware of their hypertension. We observed a wider pulse pressure among men compared to women in all groups. The pulse pressure was essentially similar between urban and rural residents regardless of hypertension and treatment status (Table 3).

**FIGURE 1:**
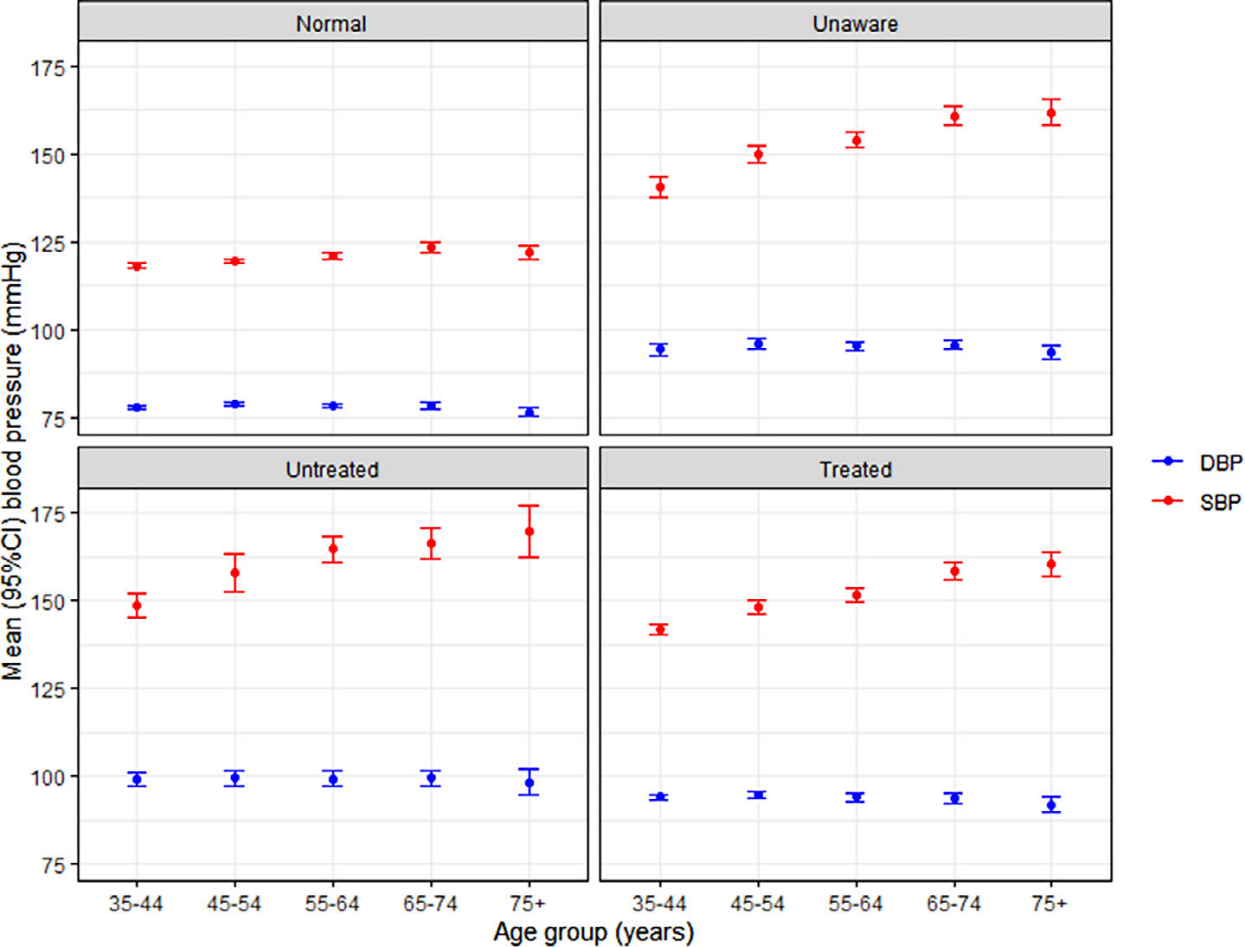
Blood pressure level by hypertension treatment status and by age group. BP level in respective groups is represented as mean ± 95% confidence interval of the mean. Systolic BP increased with age. In all groups, the youngest age category had significantly lower systolic BP compared to other age categories in all groups. Among Unaware and Treated groups, respectively, those aged 65−75 and ≥75 years had significantly higher systolic BP than other age categories. Systolic BP was similar across age groups (apart from in the youngest) in Untreated individuals. Diastolic blood pressure did not differ by age in respective groups.

**FIGURE 2:**
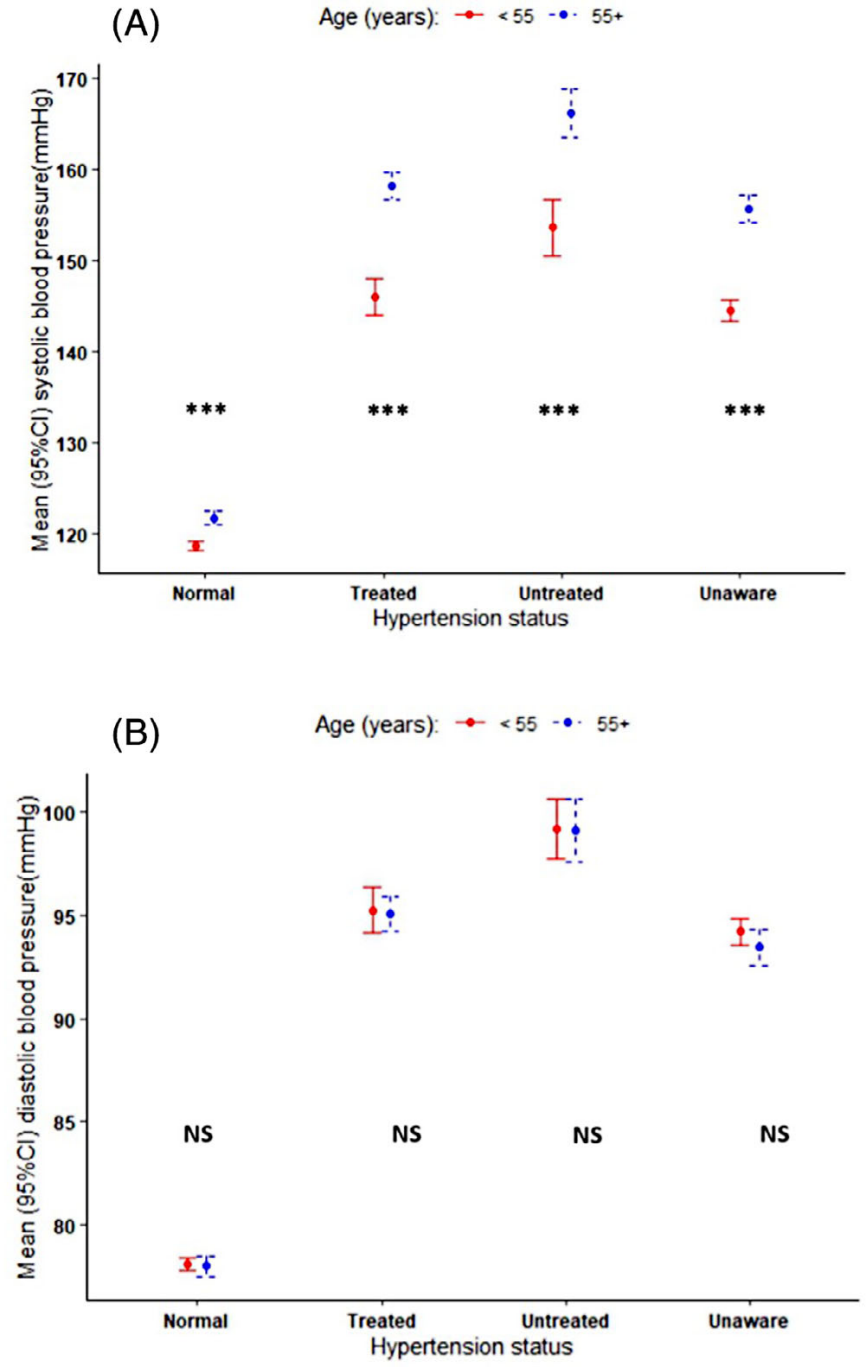
Blood pressure level stratified by age group (<55 years vs. ≥55 years) and by hypertension treatment status. (A) Systolic blood pressure level; (B) Diastolic blood pressure level. Blood pressure estimates in respective groups is represented as mean ± 95% confidence interval of the mean. NS, no significant difference; ^***^ denotes significant difference. Number of participants by age category: <55 years: Normal = 4962 (63.0%); Untreated = 293 (4.5%); Treated = 867 (13.4%); Unaware = 1229 (19.1%). ≥55 years: Normal = 807 (29.7%); Untreated = 242 (8.9%); Treated = 927 (34.1%); Unaware = 744 (27.4%).

We observed an increasing SBP with age in all groups with hypertension (Figure 1). Those unaware of their hypertension in the youngest age group had significantly higher mean SBP compared to the other groups. As expected, there was widening pulse pressure with increasing age in all groups with hypertension. When we stratified blood pressure by age categories (<55 years vs. ≥55 years), we observed significantly higher mean SBP in all hypertension categories in those aged ≥55 years. This was not observed for DBP apart from among those untreated for their hypertension (Figure 2A,B and Table S1).

## 4 DISCUSSION

In this nationally representative survey, hypertensive patients who were not receiving treatment had the highest mean blood pressure, followed by patients undergoing treatment and those who were unaware of their hypertension.

The association between elevated BP, especially SBP, and cardiovascular risk is well established^3^, ^18^, ^19^, ^20^, ^21^ and hence the very high prevalence of elevated BP in our population is a cause for concern. There may be ongoing sub‐clinical organ damage which either now, or in the future, will manifest as overt cardiovascular disease in the form of atherosclerosis, stroke, ischemic heart disease or kidney disease.

The need for improved awareness and surveillance of hypertension is underscored by the fact that 58.3% of hypertensives were not receiving treatment; nearly half of whom were unaware that they had high BP. The greatest public health benefit would derive from better identification of patients with hypertension and a successful treatment programme.^22^

However, we found that patients receiving treatment still maintained high BP levels. Excluding the possibility that treatment exacerbates the problem, the most likely explanation is that treatments are only allocated to patients with extremely high BP and the treatment is only partially effective. A study of older persons (aged 60−69 years) in the United Kingdom found significantly higher BP levels among individuals receiving treatment compared to those not receiving treatment.^23^ In Peru however, those unaware of their hypertension had the highest mean BP, followed by the treated, then those aware but not receiving treatment.^24^ Treatment failure in our study could be explained by several patient‐related factors including poor adherence which might be related to side effects and local beliefs or misconceptions around hypertension,^25^, ^26^, ^27^ or a lack of awareness of the serious risks to their future health. Medication adherence is a vital component to treatment success which has been previously evaluated in The Gambia and found to be sub‐optimal.^28^ Other factors potentially influencing BP control are lifestyle factors, such as weight control, dietary habits, and physical activity levels.^29^, ^30^, ^31^ Although these were not assessed in the present study, these factors may be more highly prevalent among treated hypertensive patients compared to the other groups.

Most of the available evidence is extrapolated from treatment of North Americans Blacks. This may not be justified due to differences in cardiovascular risk, socio‐economic status, and response to antihypertensive treatment between North American Blacks and other Blacks especially native Africans.^32^ To the best of our knowledge there have only been two multi‐country studies conducted exclusively on sub‐Saharan African populations. These are the newer versus older antihypertensive agents in African hypertensive patients (NOAAH) trial^33^ and Comparison of Three Combination Therapies in Lowering Blood Pressure in Black Africans (CREOLE) clinical trials.^34^ The NOAAH trial found a combination of amlodipine/valsatan to be more effective at controlling SBP compared to bisoprolol/hydrochlorothiziade in native Africans. In the CREOLE study, amlodipine plus either hydrochlorothiazide or perindopril was more effective than perindopril plus hydrochlorothiazide at lowering BP at 6 months. From baseline, the reduction in SBP in the latter study were −3.14 mmHg (95% CI, −5.90 to −0.38; *p* = .03), −3.00 mmHg (95% CI, −5.81 to −0.20 mmHg; *p* = .04), and −0.14 mmHg (95% CI, −2.90 to 2.61; *p* = .92), respectively.

The quality of medicines dispensed to patients may also be a major factor leading to sub‐optimal care. In a quality evaluation of seven routinely used cardiac drugs in 10 sub‐Saharan countries, two of the common antihypertensive agents (amlodipine and captopril) were found to be underdosed with the lowest ratio of measured to expected content of active ingredient of 49.2%.^35^ In sub‐Saharan Africa, factors such as shortage of medicines, high cost of medicines, busyness of doctors due to high patient load, lack of appropriate education and counselling services, poor patient‐provider interaction, and long waiting times have been reported as possible factors.^36^, ^37^

There should be more localized data to understand determinants of and barriers to treatment uptake to adapt public health interventions to the local context. As reported elsewhere, these factors include patients being worried about the need to take medication for life, perceived side effects of drugs, loss to follow‐up, and inadequate counselling from physician at the time of diagnosis.^38^, ^39^, ^40^, ^41^ Our study also highlights the need to identify factors leading to non‐treatment of patients with hypertension. A lot of patients rely on traditional treatment methods whose effect has not been evaluated. There are suggestions that patients who are physically active, on a low salt diet, and current smokers had an increased chance of being untreated,^42^ which has not been evaluated in this setting.

Our study found a high proportion with undiagnosed hypertension with concerning levels of blood pressure. As reported in a separate analysis, young people, those without comorbidities or risk factors such as smoking are disproportionately under‐diagnosed.^22^ In young people and those without risk factors, perceptions, health‐seeking behaviors, and level of contact with healthcare providers are therefore lower and hence are less likely to be screened and diagnosed. This therefore calls for a better strategy of identifying hypertension in this population for targeted intervention.

The Gambia and other countries in the sub‐region should consider the implementation of the World Health Organization (WHO) HEARTS technical package,^43^ which enables and promotes a strengthened, responsive, and resilient primary health care system. The HEARTS program which has been successfully implemented elsewhere particularly in the Americas,^44^, ^45^ ensures that patients’ cardiovascular health is managed in a comprehensive way through providing counselling about a healthy lifestyle, using evidence‐based treatment protocols, ensuring access to essential medicines and technologies, and using a risk‐based team approach, a monitoring and evaluation system using simple patient registries and also a team‐based approach through task‐shifting to care delivery.^46^

The main strength of our study is the use of an age and sex‐standardized analysis of a nationally representative sample. However, the findings should be considered with some limitations. The results are not generalizable to the general population given we only included adults aged 35 years and above. Furthermore, we used only a cross‐sectional measurement of BP when current clinical approaches require several measurements at different timepoints. Our assessment also only considered pharmacological treatment and did not include other lifestyle approaches.

## 5 CONCLUSION

The present analysis shows concerningly high levels of blood pressure in all groups with hypertension. This is particularly concerning in patients undergoing BP treatment and calls for reinforcing treatment adherence, revisiting current pharmacological treatment guidelines as well as emphasising lifestyle interventions in patient management. A culturally sensitive comprehensive programme to improve treatment allocation of untreated cases as well as detection of undiagnosed cases should be developed and implemented.

## AUTHOR CONTRIBUTIONS

Matthew J. Burton acquired the funding for this study. Modou Jobe, Islay Mactaggart, Abba Hydara, Andrew M. Prentice, and Matthew J. Burton conceived the study. Modou Jobe, Islay Mactaggart, and Min J. Kim curated and validated the data. Modou Jobe, Islay Mactaggart, Suzannah Bell, Abba Hydara, Andrew M. Prentice, and Matthew J. Burton designed and implemented the study. Omar Badjie supported the implementation of the study. Modou Jobe conducted the literature review, with support from Andrew M. Prentice. Modou Jobe and Gaetan Brezesky Kotanmi performed the analysis. Andrew M. Prentice, Matthew J. Burton, Min J. Kim, Islay Mactaggart, Omar Badjie and advised on analysis and interpretation of the data. Modou Jobe and Gaetan Brezesky Kotanmi drafted the manuscript. Islay Mactaggart, Suzannah Bell, Min J. Kim, Abba Hydara, Omar Badjie, Andrew M. Prentice, and Matthew J. Burton revised the manuscript. All authors had final responsibility for the decision to submit for publication.

## CONFLICT OF INTEREST STATEMENT

The authors declare no conflicts of interest.

## Supporting information

Supporting Information

## Data Availability

Survey content is available upon request. For any data requests, please contact Islay Mactaggart (Islay.Mactaggart@lshtm.ac.uk).
