# Supplementary material for: Blood pressure and the hypertension care cascade in The Gambia: Findings from a nationwide survey
Source: J Clin Hypertens (Greenwich). 2024 Apr 2;26(5):563–72. doi: 10.1111/jch.14806 (PMC11088434; doi:10.1111/jch.14806)
Supplement: Supplementary file 1 — Supporting Information [file JCH-26-563-s001.docx]

Supplementary table 1: Blood pressure level stratified by age group (<55 years vs ≥55 years) and by hypertension treatment status.

| **Age category*** | **Hypertension status** | **Blood pressure** |
| --- | --- | --- |
|  | **Systolic** | |
| <55 years | Normal | 118.7 (118.2-119.1) |
|  | Untreated | 153.5 (150.4-156.7) |
|  | Treated | 145.9 (144.0-147.9) |
|  | Unaware | 144.5 (143.4-145.6) |
| ≥55 years | Normal | 121.7 (120.9-122.5) |
|  | Untreated | 166.1 (163.4-168.9) |
|  | Treated | 158.2 (156.6-159.7) |
|  | Unaware | 155.6 (154.1-157.1) |
|  | **Diastolic** | |
| < 55 years | Normal | 78.1 (77.8-78.4) |
|  | Untreated | 99.2 (97.7-100.6) |
|  | Treated | 95.2 (94.1-96.3) |
|  | Unaware | 94.2 (93.6-94.9) |
| ≥55 years | Normal | 78.0 (77.5-78.5) |
|  | Untreated | 99.1 (97.5-100.6) |
|  | Treated | 95.0 (94.2-95.9) |
|  | Unaware | 93.4 (92.6-94.3) |

*Number of participants by age category:

<55 years: Normal=4962 (63.0%); Untreated=293 (4.5%); Treated=867 (13.4%); Unaware=1229 (19.1%)

≥55 years: Normal=807 (29.7%); Untreated= 242 (8.9%); Treated= 927 (34.1%); Unaware=744 (27.4%)
